# Regulation of nutrient metabolism by nuclear receptor/FGF signaling pathways

**DOI:** 10.1186/1753-6561-6-S3-O16

**Published:** 2012-06-01

**Authors:** Steven A Kliewer, David J Mangelsdorf

**Affiliations:** 1 Department of Pharmacology and the Howard Hughes Medical Institute, University of Texas Southwestern Medical Center, Dallas, TX 75390, USA

## 

The regulation of metabolism in fed and fasted states is governed by hormonal and nutrient-derived signals that are mediated in part by nuclear receptors. Just as insulin and glucagon help the body store and mobilize energy through their membrane receptors, nutrient-derived lipids activate their cognate nuclear receptors (e.g., FXR and PPARs) to govern transcriptional programs involved in energy storage and utilization during times of nutrient excess and privation. Recent work has revealed that many of the actions of these nuclear receptors are mediated by two atypical fibroblast growth factors (FGF19 and FGF21) that function as endocrine-like hormones. The characterization of these pathways in normal and metabolic disease animal models has led to an understanding of their physiologic and pharmacologic mechanism of action.

